# Analysis of the sheep (Ovis aries) vaginal microbiota preceding spontaneous abortion: a pilot study

**DOI:** 10.1099/acmi.0.001005.v3

**Published:** 2025-07-25

**Authors:** Lucille C. Jonas, Stephan Schmitz-Esser, Curtis R. Youngs

**Affiliations:** 1 Department of Animal Science, Iowa State University, Ames, IA, USA; 2 Microbiology Graduate Program, Iowa State University, Ames, IA, USA

**Keywords:** *Campylobacter*, ewe, *Histophilus*, livestock, reproduction

## Abstract

Little is known regarding the vaginal microbiota of sheep that undergo spontaneous abortions. The aim of this pilot study was to characterize, using 16S rRNA gene sequencing and shotgun metagenomics, the vaginal microbiota throughout the gestation of two ewes (Ewe1 and Ewe2) that spontaneously aborted. To achieve this, weekly vaginal swabs were collected from the ewes prior to breeding until pregnancy testing; thereafter, biweekly swabs were collected until the spontaneous abortion occurred. Based on the 16S rRNA sequencing data, Ewe1’s vaginal microbiota, overall, contained high abundances of *Histophilus* (12.9% relative abundance), *Staphylococcus* (10.8% relative abundance) and Unclassified *Pasteurellaceae* (8.7% relative abundance). Most notable was the high abundance of *Campylobacter* following the abortion in Ewe1’s vaginal microbiota. Ewe2’s vaginal microbiota was characterized by high abundances of *Pasteurella* (41.7% relative abundance) throughout gestation. Shotgun metagenomic sequencing produced two high-quality metagenome-assembled genomes (MAGs), identified as *Campylobacter jejuni* and *Histophilus somni*. The *C. jejuni* MAG had 99.95% average nucleotide identity to the most abundant sheep abortive * C. jejuni* clone in the USA. The *H. somni* MAG was most similar to a pathogenic *H. somni* strain and contained genes that contribute to serum resistance and sialic acid utilization. The results presented here demonstrate the need for continued research into the vaginal microbiota, specifically to identify potential predictors of spontaneous abortion.

## Data Summary

The authors confirm that all supporting data, code and protocols have been provided within the article. The raw sequencing data were deposited in the NCBI-SRA under BioProject accession number PRJNA1043118. The BioSample accessions for the metagenome shotgun sequencing data are as follows: Ewe1 (Week 13: SAMN46514396, Week 15: SAMN46514395), Ewe2 (Week 14: SAMN46514397, Week 16: SAMN46514398). The metagenome-assembled genomes produced in this study are also available under BioProject PRJNA1043118.

## Introduction

Recently, we documented dynamic changes in the sheep (*Ovis aries*) vaginal microbiota throughout gestation, likely occurring in response to hormonal fluctuations during pregnancy [1]. This study improved our understanding of the vaginal microbiota of ewes with successful pregnancies. However, it may not be reflective of the vaginal microbiota in ewes with abnormal pregnancies, as previous research reported connections between reproductive status and the sheep vaginal microbiota [2,5]. Infectious abortions have considerable economic impacts in sheep production systems [6], yet very little is known about the vaginal microbiota in ewes with spontaneous abortions. Here, we present an exploratory pilot study characterizing the vaginal microbiota throughout the gestation of two ewes that subsequently aborted. The aim of this analysis was to describe the composition of the ewe vaginal microbiota with 16S rRNA gene amplicon sequencing and characterize abundant community members with shotgun metagenomics. Our results increase the understanding of the vaginal microbiota as a reproductive performance indicator or diagnostic tool of abnormal pregnancies.

## Methods

### Ethics statement

All animal procedures were conducted after approval by the Iowa State University Institutional Animal Care and Use Committee (protocol no. 21-141).

### Sample collection and processing

The two ewes examined in this analysis (Ewe1 and Ewe2) were part of a larger cohort investigated previously [1] and subjected to the same management and sampling practices. Ewe1 was in poor body condition and spontaneously aborted prior to sampling week 15. Ewe2 was asymptomatic but aborted prior to sampling week 18. This resulted in 14 vaginal swabs collected from Ewe1 and 17 vaginal swabs from Ewe2.

Vaginal swabs from Ewe1 and Ewe2 were processed as previously described; DNA was extracted using the DNeasy PowerLyzer PowerSoil kit (Qiagen, Germantown, MD, USA) [1]. Using an Illumina MiSeq platform, the V4 region of the 16S rRNA gene was sequenced to analyse the vaginal microbiota [1]. Additionally, metagenome shotgun sequencing was performed using Illumina NovaSeq 150 bp paired-end sequencing on two samples from both ewes (sampling weeks 13 and 15 for Ewe1 and weeks 14 and 16 for Ewe2) to investigate in greater detail abundant bacteria of the vaginal microbiota surrounding the ewes’ spontaneous abortions.

### Sequence analysis

Analysis of the 16S rRNA gene amplicon sequences was performed as previously described [1]. Metagenome shotgun sequencing data were initially checked for quality by using FastqC [7]. Adapter trimming and initial quality control were conducted with BBduk [8]. Sequences were then mapped to the reference sheep genome (ARS-UI_Ramb_v3.0, accession: GCA_016772045.2) [9] to separate microbial from host reads using BBmap [8]. MetaPhlAn4 was used to determine the microbial taxonomic composition of metagenome sequencing reads [10]. A co-assembly of sequences from all samples was conducted with Megahit to create contiguous sequences (contigs) [11]. Contigs were binned into metagenome-assembled genomes (MAGs) with Metabat2 [12]. The quality of the MAGs was assessed with CheckM [13]. Identification of the MAGs was initially conducted with tetra correlation searches using the GenomesDB reference database on JspeciesWS [14]. Pairwise average nucleotide identities (ANIs) were also conducted with relevant reference genomes using JspeciesWS [14]. Using the Bacterial and Viral Bioinformatics Resource Center (BV-BRC) platform, the genome annotation tool was used to annotate the MAGs with RASTtk, and the BV-BRC Proteome Comparison Service tool was used to compare the MAGs with relevant reference genomes using blastP [15,16].

## Results

### Composition of the vaginal microbiota

**Fig. 1.**
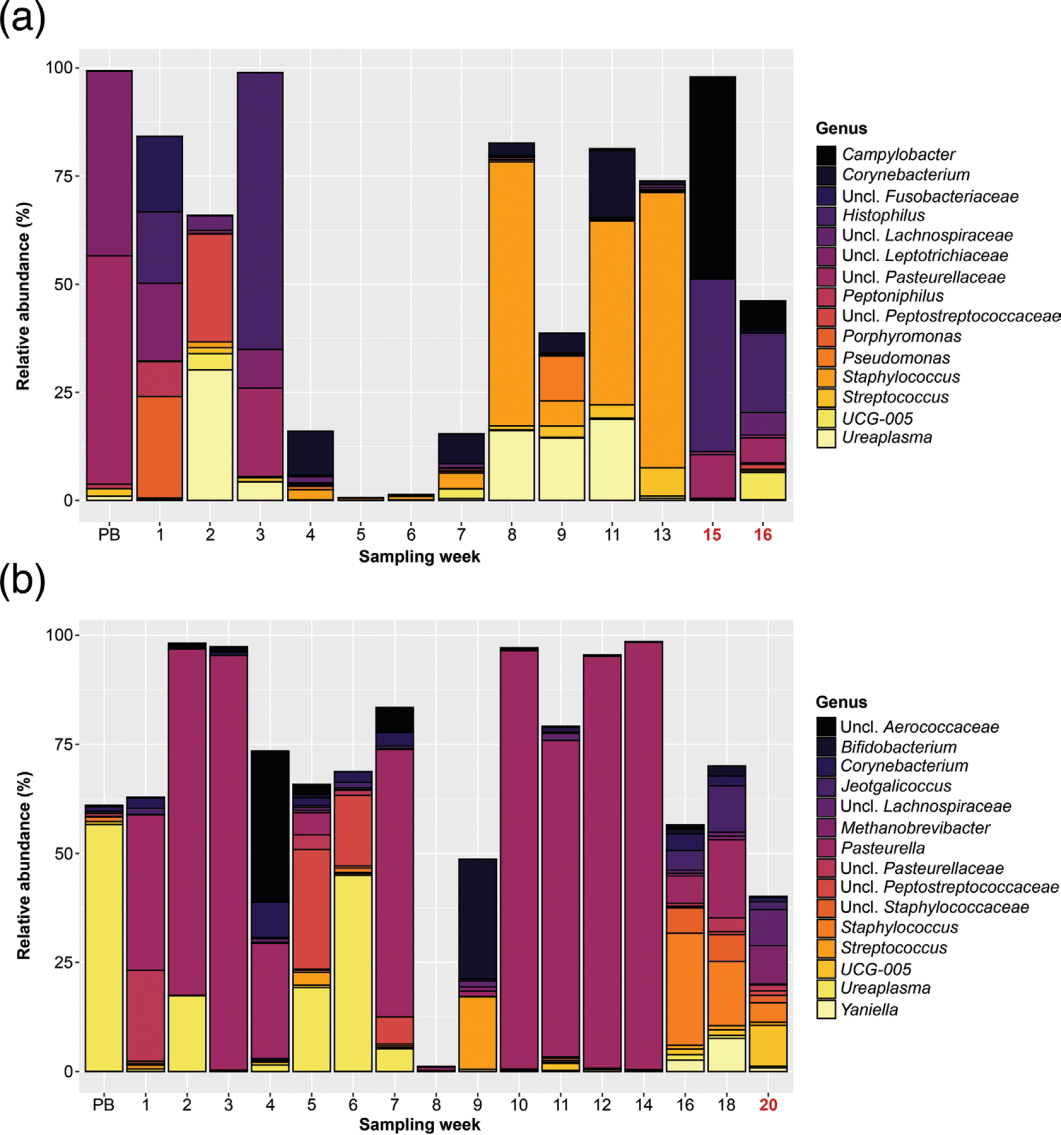
Relative abundance of the 15 most abundant bacterial genera in the vaginal microbiota of (a) Ewe1 and (b) Ewe2 from pre-breeding, during gestation, and following the spontaneous abortion based on 16S rRNA amplicon sequencing (post-abortion sampling weeks are highlighted in red). Unclassified is abbreviated as ‘Uncl.’.

Following 16S rRNA gene amplicon sequencing, the 31 samples contained a total of 529,147 sequences. At the genus level, the vaginal microbiota of Ewe1 and Ewe2 differed substantially and varied highly between sampling time points (Fig. 1). The most abundant genera in Ewe1’s samples (Fig. 1a) were *Histophilus*, *Staphylococcus* and an unclassified genus in the *Pasteurellaceae* family, making up 12.9, 10.8 and 8.7% of relative abundance, respectively. *Campylobacter* was found in low abundance (0.1%) in the pre-breeding through week 9 samples (average relative abundance 0.01%) but in higher abundances during sampling weeks 11 (0.4%) and 13 (0.1%). Vaginal samples collected post-abortion exhibited the highest abundance of *Campylobacter* (week 15: 46.7%, week 16: 6.9%). Of note in Ewe1 are samples with relatively low abundance of any of the 15 most abundant genera of its vaginal communities. Specifically, in Ewe1’s week 5 timepoint, Unclassified *Rhodobacteraceae* (11.3% relative abundance), Unclassified *Alphaproteobacteria* (4.6% relative abundance) and Unclassified *Gammaproteobacteria* (4.2% relative abundance) were the three most abundant genus-level classifications. Along with this, *Dokdonella* (*Rhodobacteraceae*, 8.8% relative abundance), *Runella* (*Spirosomaceae*, 4.2% relative abundance) and AKYH767_ge (*Sphingobacteriales*, 3.8% relative abundance) were the three most abundant genus-level classifications in Ewe1’s week 6 sample. Starting at sampling week 7, there appears to be a shift in the vaginal microbiota of Ewe1 to higher abundances of *Staphylococcus* preceding the abortion. The relative abundance of *Staphylococcus* in Ewe1 increased to 61.0% in sampling week 8 and was 42.5 and 63.7% in weeks 11 and 13, respectively.

Ewe2’s vaginal microbiota (Fig. 1b) appeared to be less rich and was dominated by *Pasteurella* (41.7% relative abundance), *Ureaplasma* (7.8% relative abundance) and an unclassified genus in the *Peptostreptococcaceae* family (3.5% relative abundance). In Ewe2, sampling week 8 had a low abundance of the overall top 15 genera of its vaginal communities; instead, this sample contained Unclassified *Rhodobacteraceae* and Unclassified *Alphaproteobacteria*, which made up 10.5 and 4.6% of the sample’s relative abundance, respectively. Similar to Ewe1, the relative abundance of *Staphylococcus* increased prior to the abortion to 25.7 and 14.7% in Ewe2’s week 16 and 18 samples, respectively.

### Metagenomic investigation of the ewe vaginal microbiota

One limitation of 16S rRNA gene sequencing is that it does not provide functional insights or species-level taxonomic resolution. Therefore, we applied metagenome shotgun sequencing, which generated 1.736 billion sequences. After quality control, 1.586 billion sequences remained, of which 95% mapped to the sheep genome and thus were excluded, leaving 78,984,420 reads for further analysis. Taxonomic profiling of the metagenome sequencing data mirrored what was seen within the 16S rRNA amplicon data.

Ewe1’s taxonomic profile was largely richer than that of Ewe2. The most abundant species in Ewe1’s week 13 sample was an unclassified *Micrococcaceae* species (GGB42949_SGB60172), which made up 16.6% of the classified bacterial sequences. This was followed by *Staphylococcus equorum* and *Jeotgalicoccus coquina*, making up 16.4 and 13.6% of classified bacterial sequences. The most abundant bacterial species in Ewe1’s week 15 sample was *Campylobacter jejuni*, making up 65.2% of the classified sequences, followed by *Histophilus somni* (34.6% relative abundance).

The classified bacterial sequences in Ewe2’s week 14 sample were exclusively defined as *Pasteurella multocida*. The same unevenness was observed in Ewe2’s week 16 sample; however, these sequences were solely classified as *S. equorum*.

The unmapped microbial sequences were co-assembled into 907,504 contigs, with an average length of 457 bp and an N50 of 470 bp. MAG binning from the contigs produced two high-quality bacterial MAGs (Table 1).

**Table 1.** Overview of the MAGs derived from the ewes that aborted

	*C. jejuni* MAG	*H. somni* MAG
**Assembly size (bp**)	1,607,690	2,018,570
**CheckM completeness (%)**	99.57	99.16
**CheckM contamination (%)**	0.11	0.80
**Number of contigs**	21	51
**G+C (mol%)**	30.5	37.0
**NCBI accession number**	GCA_048159425.1	GCA_048159405.1
**Closest relative** **(% ANI)**	*C. jejuni* IA3902 NC_017279.1 (99.95%)	*H. somni* 2336 NC_010519.1 (98.23%)

One MAG was identified as *C. jejuni* and was of MLST 8. The *C. jejuni* MAG had an ANI of 99.95% (99.78% overlap) to the major sheep abortive *C. jejuni* clone in the USA [17,18]. The second MAG was most similar to pathogenic *H. somni* strain 2336 [19], with an ANI of 98.23% (90.25% overlap). The presence of immunoglobulin binding proteins IbpA (84.2% amino acid identity to BAC78649.1) and IbpB (94.9% amino acid identity to BAC78648.1) within the *H. somni* MAG is associated with virulence [20,21]. Proteins involved in sialic acid catabolism [NanA (WP_075293756), NanK (WP_012340290) and NanE (WP_249962365); 99, 99 and 98%, amino acid identity, respectively] were found in the *H. somni* MAG, along with potential capacity for lipooligosaccharide (LOS) sialylation based on the presence of NeuA (99.5% amino acid identity to WP_075293765) and SiaA (98.7% amino acid identity to WP_012340571).

## Discussion

The vaginal microbiota has been connected with animal reproductive performance [2,4, 22, 23]. A recent study from our group defined changes in ewe vaginal microbiota during gestation, illustrating a potential relationship between animal hormonal status and vaginal microbiota [1]. The exploratory pilot study presented here provides the first insights into the vaginal microbiota in ewes with spontaneous abortions. Although this pilot study used samples from only two ewes, we generated a unique and novel dataset due to the repeated sampling throughout gestation. The detailed characterization of the vaginal microbiota, not only over time but also through metagenome shotgun sequencing, has not been performed previously with ewes that undergo spontaneous abortions, enabling a unique contribution to the scientific literature. Results from the present study ideally need to be verified with similar studies utilizing more animals, although such studies are difficult to conduct due to the inability to predict *a priori* ewes that will spontaneously abort.

Ewe1’s abortion is likely connected to the presence of *C. jejuni* in its vaginal microbiota. *C. jejuni* is a leading cause of abortions in sheep flocks in the USA [24]. Because the *C. jejuni* MAG obtained was virtually identical (99.95% ANI) to the most abundant US sheep abortive *C. jejuni* clone [25], it can be assumed that this MAG is involved in Ewe1’s abortion. This assumption is also supported by the increase in relative abundance of *Campylobacter* in Ewe1’s vaginal microbiota at sampling week 11, which potentially reflects the incubation period of *C. jejuni* preceding abortion.

*H. somni*, also present in Ewe1’s vaginal microbiota, is historically regarded as commensal in animal mucous membranes; however, it is also known to cause a variety of diseases in ruminants, such as bovine respiratory disease, septicaemia, abortion or myocarditis [26]. The function of *Histophilus* in the sheep vaginal microbiota is unclear. *Histophilus* was abundant in the healthy ewe vaginal microbiota [1,3] but was also found more abundant in ewes who failed to conceive or aborted [5,27]. The *H. somni* MAG contained IbpA and IbpB, which are linked to serum resistance and cytotoxicity [20,28]. Genes linked to sialylation of LOS (*neuA*, *siaA* and *nanAKE*) were also found in the *H. somni* MAG and also contribute to host immune evasion [29]. The interpretation of these findings in the *H. somni* MAG is complicated due to the lack of complete vaginal *H. somni* reference genomes in NCBI GenBank. Future isolation and genome sequencing of vaginal *H. somni* isolates will provide more insights into the prevalence of certain virulence factors and the differentiation of commensal and virulent * H. somni* isolates.

Ewe2’s vaginal microbiota exhibited a high degree of variability. Beginning at the first week of breeding (sampling week 1), *Pasteurella* made up the majority of the sequences within vaginal communities, potentially before the start of pregnancy. Metagenomic sequencing revealed that the vaginal microbiome of Ewe2 contained *P. multocida* specifically. *P. multocida* can cause a variety of pathologies, including haemorrhagic septicaemia, pneumonia and atrophic rhinitis [30]. In the case of Ewe2, the vaginal microbiota is possibly a reflection of the ewe’s poor health status, demonstrating it was not suitable for pregnancy given the very high abundance of *Pasteurella* in the vaginal microbiota. However, the vaginal microbial profiles of more ewes that undergo spontaneous abortions need to be examined to identify patterns.

While the vaginal microbiota of Ewe1 and Ewe2 exhibit significant differences, several common features are evident. Examining the 16S rRNA sequencing data, both ewes appear to have a shift in their vaginal microbiota, during which there is very low coverage of the most abundant genera in either ewe. This occurred at sampling week 5 for Ewe1 and sampling week 8 for Ewe2. A similar shift was seen in healthy ewes with successful pregnancies from the same flock [1]. In the healthy population, the ewe vaginal microbiota increased in taxa richness and evenness, peaking at gestational week 8. We hypothesize that the observed shift in microbial community structure may be related to pregnancy-associated hormonal changes occurring within the ewe during this week of gestation. However, validation of this requires additional investigation. Following this shift, both ewes have increased abundances of *Staphylococcus* in their vaginal microbiota leading up to both abortions. In both cases, *S. equorum* was specifically identified within the shotgun sequencing data. It is unclear if the *Staphylococcus* is related to the abortions, as *Staphylococcus* also increased in abundance during late gestation of sheep with healthy pregnancies [1].

## Conclusion

Results of this analysis illustrate a connection between the vaginal microbiota and reproductive failure, further emphasizing the need for continued investigation. The vaginal microbiota of ewes that experienced spontaneous abortions was distinct from that of reproductively healthy ewes. In addition, the metagenome shotgun sequencing allowed preliminary functional insights into two MAGs and provided higher taxonomic resolution for abundant members of the vaginal microbiota. Additional research is needed to validate the results presented here, specifically gathering microbiota data on more ewes that experience spontaneous abortion.

## Abbreviations

ANI: average nucleotide identity
BV-BRC: Bacterial and Viral Bioinformatics Resource Center
LOS: lipooligosaccharide
MAG: metagenome-assembled genome
